# Clinical significance of serum anti-granulocyte–macrophage colony-stimulating factor autoantibodies in patients with sarcoidosis and hypersensitivity pneumonitis

**DOI:** 10.1186/s13023-020-01546-x

**Published:** 2020-09-29

**Authors:** Kanako Katayama, Masaki Hirose, Toru Arai, Kazuyoshi Hatsuda, Kazunobu Tachibana, Reiko Sugawara, Chikatoshi Sugimoto, Takahiko Kasai, Masanori Akira, Yoshikazu Inoue

**Affiliations:** 1 Department of Internal Medicine, National Hospital Organization Kinki-Chuo Chest Medical Center, Sakai City, Osaka Japan; 2 Clinical Research Center, National Hospital Organization Kinki-Chuo Chest Medical Center, Sakai City, Osaka Japan; 3 Department of Pathology, National Hospital Organization Kinki-Chuo Chest Medical Center, Sakai City, Osaka Japan; 4 Department of Radiology, National Hospital Organization Kinki-Chuo Chest Medical Center, Sakai City, Osaka Japan

**Keywords:** Autoantibody, Granulocyte–macrophage colony-stimulating factor, Hypersensitivity pneumonitis, Pulmonary alveolar proteinosis, Sarcoidosis

## Abstract

**Background:**

Anti-granulocyte–macrophage colony-stimulating factor autoantibody (GMAb) has been recognized as a diagnostic biomarker for autoimmune pulmonary alveolar proteinosis (aPAP). The aims of this study were to know the incidence of increased level of serum GMAb in granulomatous lung diseases (sarcoidosis and hypersensitivity pneumonitis [HP]) and to clarify the role of GMAb. Consecutive individuals diagnosed with sarcoidosis (n = 92) and HP (n = 45) at National Hospital Organization Kinki-Chuo Chest Medical Center were retrospectively analyzed. We measured serum GMAb levels at the diagnosis. Cut-off values of GMAb discriminating aPAP (n = 110) from healthy controls (n = 31) were determined by receiver operating characteristic (ROC) curve analysis. We compared the clinical features of sarcoidosis and HP patients with GMAb levels above the cut-off value (“Elevated-GMAb”) with those of patients whose GMAb levels below the cut-off value (“Low-GMAb”). Radiological and pathological findings in elevated-GMAb patients were re-evaluated to elucidate the role of GMAb in granulomatous lung diseases.

**Results:**

Analysis of ROC indicated a sensitivity and specificity of 100% at GMAb level of 3.33 μg/mL for discriminating aPAP from healthy controls (area under curve = 1.000, *p* < 0.0001). The percentages of elevated-GMAb sarcoidosis and HP patients were 5.4% (n = 5) and 11.1% (n = 5), respectively. The number of comorbid sarcoidosis and HP patients with aPAP was two and one, respectively. Elevated-GMAb sarcoidosis patients presented with significantly higher serum levels of Krebs von den Lungen (KL)-6, surfactant protein-D (SP-D), lactate dehydrogenase, and the requirement of systemic corticosteroid therapy. Elevated-GMAb HP patients demonstrated older age, higher serum KL-6, SP-D, carcinoembryonic antigen, and cytokeratin fragment 21-1 levels, and a higher percentage of lymphocytes in bronchoalveolar lavage than low-GMAb patients. A subset of patients presented with radiological and pathological findings characteristic of aPAP.

**Conclusions:**

We demonstrated the percentage of elevated-GMAb sarcoidosis and HP patients who presented with several features suggestive of aPAP. Elevated-GMAb sarcoidosis and HP patients without definitive aPAP diagnosis may have subclinical or early-stage aPAP and may not necessarily indicate false positives. Upon diagnosis of sarcoidosis or HP, measurement of GMAb may be useful in detecting possible comorbidity of subclinical or early-onset aPAP.

## Introduction

Pulmonary alveolar proteinosis (PAP) is a rare disorder characterized by the accumulation of surfactant lipids and proteins in alveolar spaces [1–3]. Kitamura et al. detected neutralizing anti-granulocyte–macrophage colony-stimulating factor autoantibody (GMAb) in serum and bronchoalveolar lavage from autoimmune pulmonary alveolar proteinosis (aPAP) patients [4, 5]. Insufficient maturation of pulmonary monocyte/macrophage populations in the presence of GMAb with neutralizing capacity of granulocyte–macrophage colony-stimulating factor (GM-CSF) is considered to cause aPAP [2, 3, 5, 6]. In aPAP, GMAb is pathogenic and thus acts as a diagnostic biomarker. We diagnosed aPAP using our serum cut-off value of GMAb to discriminate aPAP from normal controls and control diseases [3, 7, 8].

In antigen presentation and granuloma formation, GM-CSF plays an important role [9]. Sarcoidosis is a systemic granulomatous disorder with unknown etiology, and previous reports have suggested that its pathophysiology is an aberrant immune response driven by an unidentified antigen, and associated with macrophage/monocyte-derived cytokines such as GM-CSF [10]. Hypersensitivity pneumonitis (HP) is a granulomatous lung disease characterized by an immunological reaction to inhaled antigens [11]. It is hypothesized that environmental exposure may play a role in triggering autoimmunity in aPAP [12, 13]. Hence, some pathophysiological link between aPAP and granulomatous diseases including sarcoidosis and HP might be present.

Recently, several aPAP patients with sarcoidosis have been reported [14–18]. Verma et al. described five patients with histopathological features of both PAP and HP [19]. Nishimura et al. [20] reported that serum GMAb levels were slightly elevated in sarcoidosis patients compared with healthy controls. Relatively elevated-GMAb levels in granulomatous lung diseases such as sarcoidosis or HP may exist, but the incidence and clinical features of this condition are unknown. This study aimed to retrospectively analyze the incidence and clinical features of elevated-GMAb levels with sarcoidosis and HP.

## Patients and methods

### Patients

This study included consecutive sarcoidosis (n = 92) and HP (n = 45) patients whose serum samples were stored at diagnosis in the National Hospital Organization (NHO) Kinki-Chuo Chest Medical Centre between June 2003 and December 2017. Patients underwent bronchoscopy and/or surgical lung biopsy for the pathological diagnosis. Medical conditions of all patients were discussed and their diagnoses were established at our institutional multidisciplinary conferences. To determine the cut-off values to distinguish aPAP from healthy controls, this study also included consecutive individuals diagnosed with aPAP (n = 110) in our institute between July 1997 and September 2016, and age-/sex-matched healthy controls (n = 31). Healthy controls had no symptoms, history of major illnesses, or abnormal shadows on chest radiography during check-up at our hospital in 2017.

### Diagnostic criteria of sarcoidosis, HP and aPAP

Sarcoidosis diagnosis was based on the 2006 Diagnostic Criteria and guidelines for sarcoidosis published by the Japanese Society of Sarcoidosis and Other Granulomatous Disorders (JSSOG) [21, 22].

The diagnosis of HP was arrived at based on the following criteria [11, 23–26]: a combination of compatible symptoms (dyspnea and/or cough and/or symptoms with onset shortly after exposure and/or recurrent viral symptoms), suggestive computerized tomography (CT) findings (extensive ground glass opacities [GGOs], centrilobular nodule, peribronchovascular fibrosis, upper lobe predominant fibrosis and/or air trapping), and suggestive pathological findings (presence of granulomas, giant cells and/or mixed bronchocentric/usual interstitial pneumonia-like pattern) or compatible pathological findings (any fibrosis without a higher probability of any other disease). Patients with chronic HP were required to have pathological findings, high-resolution computed tomography (HRCT) findings, or both, indicative of fibrosis or respiratory symptoms that were present for six months or longer [23].

The diagnosis of aPAP was based on pathological and/or cytological findings of transbronchial lung biopsy, bronchoalveolar lavage (BAL), or surgical lung biopsy with both HRCT appearance and GMAb level higher than 95th percentile of healthy controls according to the diagnostic criteria of the Japanese Ministry of Health, Labour and Welfare and our recent review article [3, 27].

### Clinical findings

Data were extracted from medical records. Variables included demographic data, laboratory data, BAL fluid findings, and pulmonary function test results at sarcoidosis and HP diagnoses. We investigated the number of patients who received systemic corticosteroid treatment after sarcoidosis or HP diagnosis in our hospital.

### Measurements

At diagnosis, GMAb levels in sera were measured by the specific enzyme-linked immunosorbent assay (ELISA) methods previously reported [2, 4, 27–29], with minor modifications to monoclonal GMAb reference standard and the regression method. The monoclonal antibody used for the standard was kindly provided by EVEC, Inc. (Sapporo, Japan). We measured serum GMAb at diagnosis and follow-up latest point in GMAb-positive sarcoidosis and HP patients whose serum samples were obtained after diagnosis. In complicated cases of sarcoidosis or HP with aPAP, GMAb levels were measured at diagnosis of sarcoidosis or HP. We measured serum levels of Krebs von den Lungen (KL)-6 and surfactant protein-D (SP-D) using commercially available ELISA kits (ED046: Eisai Co., Tokyo, Japan; SP-D kit: Yamasa Co., Tokyo, Japan). Serum KL-6 and SP-D cut-off values were 500 U/mL and 110 ng/mL, respectively [2].

### Radiological and pathological evaluation

We re-evaluated radiological and pathological findings related to PAP in elevated-GMAb sarcoidosis and HP patients without complication of aPAP. All re-evaluated HRCT scans and biopsies were performed at sarcoidosis or HP diagnosis. The features on HRCT scans suggestive of PAP were GGO, interlobular septal thickening, crazing paving pattern, consolidation, geographic distribution, and subpleural sparing. Those potentially accompanying PAP were defined by traction bronchiectasis, cysts, and honeycombing. All HRCTs were reviewed and scored by a senior chest radiologist (M. A.) who was blinded to clinical information. The histologic features suggestive of PAP were eosinophilic granular materials, periodic acid-Schiff (PAS) staining-positive materials, and surfactant protein-A (SP-A) positive materials. Those potentially accompanying PAP were defined by foamy macrophages, a hyaline globule/sclerotic central core, lymphocytic infiltration, and interstitial fibrosis. Negative findings for PAP included neoplastic lesions, granulomatous lesions, neutrophilic/eosinophilic infiltration, and necrosis. All surgical lung biopsy and/or transbronchial lung biopsy slides were reviewed and scored by a senior pulmonary pathologist (T.K.) who was blinded to clinical information.

### Receiver operating characteristic (ROC) curve analysis

We performed ROC curve analysis for differentiating aPAP from the following: (1) healthy control, (2) sarcoidosis and HP patients, and (3) healthy controls, sarcoidosis, and HP patients. We used the Youden’s index for analysis. We defined patients with GMAb levels at or above the cut-off value as elevated-GMAb patients and those with GMAb levels below the cut-off value as low-GMAb patients.

### Statistical analysis

Continuous variables were reported as the median (interquartile range [IQR]) and were compared using Wilcoxon singed-rank test. Categorical variables were reported as counts (percentages) and were compared using Fisher's exact test. A *p*-value < 0.05 was considered statistically significant. All data analyses were conducted using JMP^®^ 13 (SAS Institute Inc., Cary, NC, USA).

## Results

### GMAb cut-off values for discriminating aPAP from healthy controls, sarcoidosis and HP patients, and both

Of the 92 sarcoidosis patients, two (2.1%) were complicated by aPAP. Among the 45 HP patients, one (2.2%, chronic HP) was complicated by aPAP, 39 (87%) had chronic HP, and 30 (67%) required surgical lung biopsy for diagnosis. The median follow-up period was 5.6 years (Interquartile range [IQR]: 3.6, 10.2) in sarcoidosis patients and 4.9 years (IQR: 2.4, 7.3) in HP patients. Patients’ clinical characteristics are shown in Table 1.

**Table 1:** Clinical characteristics of patients

Variables	Healthy controls	aPAP	Sarcoidosis	HP
N	31	110	92	45
Age, [IQR] years	56 [55, 59]	56 [46, 65]	59 [38, 70]	61 [53, 70]
Male, n [%]	15 [48.3]	77 [70]	39 [42.3]	24 [53.3]
Complication of aPAP, n [%]	–	–	2 [2.1]	1 [2.2]
GMAb [IQR] μg/mL	0.85 [0.52, 1.06]	40.5 [17.1, 71.8]	0.43 [0.16, 0.85]	0.87 [0.54, 1.10]

Data are expressed as the median [IQR] or number [%]

*aPAP* autoimmune pulmonary alveolar proteinosis, *HP* hypersensitivity pneumonitis, *GMAb* anti-granulocyte–macrophage colony-stimulating factor autoantibody

**Fig. 1:**
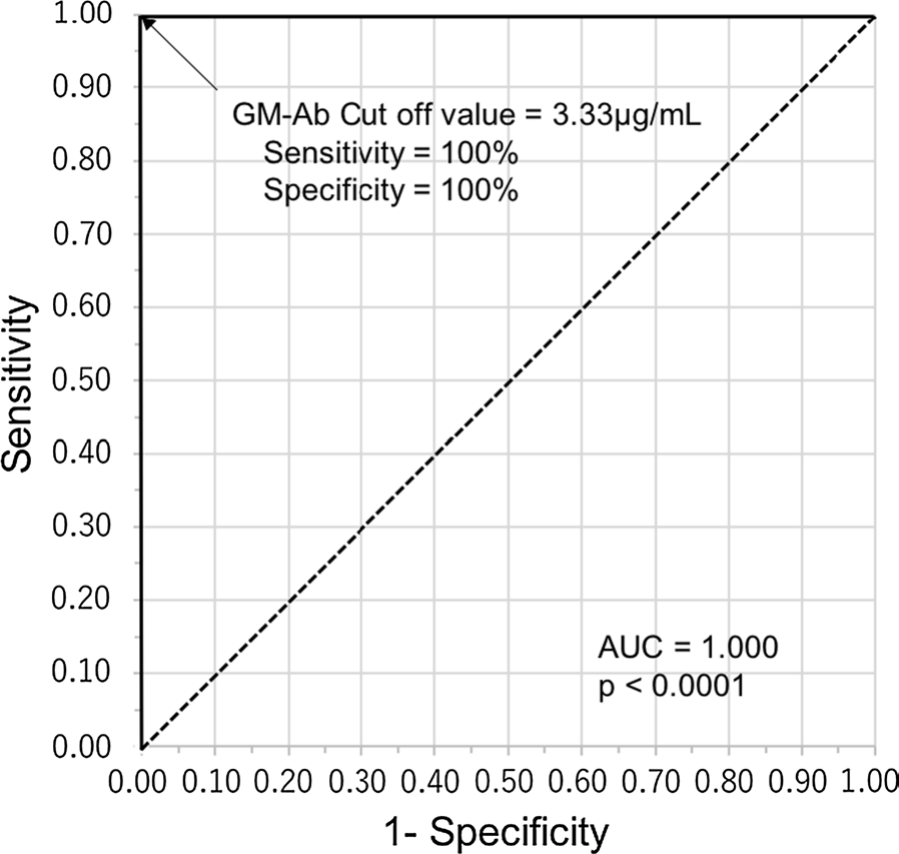
The ROC curve to discriminate aPAP from healthy controls. The best cut-off value of GMAb for discriminating aPAP (n = 110) from healthy controls (n = 31) was 3.33 μg/mL (AUC = 1.000, *p* < 0.0001, sensitivity 100%, specificity 100%). The cut-off value was determined by Youden's index. *aPAP* autoimmune pulmonary alveolar proteinosis, *GMAb* anti-granulocyte–macrophage colony-stimulating factor autoantibody

Analysis of ROC indicated a sensitivity and specificity of 100% at GMAb level of 3.33 μg/mL for discriminating aPAP from healthy controls (Area under the curve [AUC] = 1.000, *p* < 0.0001) (Fig. 1). We defined elevated-GMAb patients as those with GMAb levels $\ge$ 3.33 μg/mL. Analysis of ROC for discriminating aPAP from sarcoidosis and HP (AUC = 0.987, *p* < 0.0001) indicated a sensitivity of 100% and specificity of 94.8% at a cut-off value of 3.33 μg/mL for GMAb levels. Further, ROC analysis indicated a sensitivity of 100% and specificity of 95.8% at a cut-off GMAb value of 3.33 μg/mL for discriminating aPAP from both healthy controls and sarcoidosis and HP patients (AUC = 0.989, *p* < 0.0001).

**Fig. 2:**
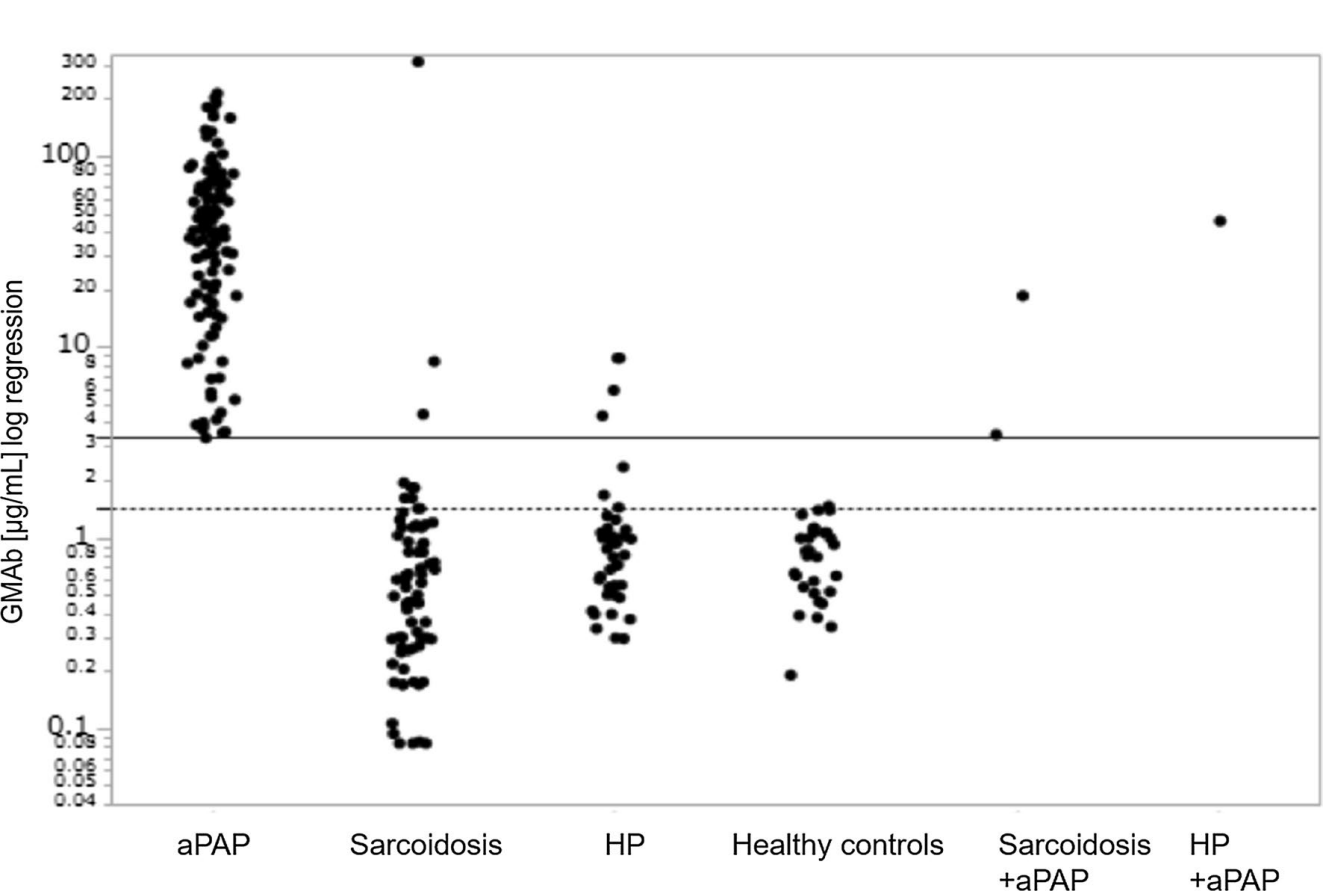
GMAb levels in serum. GMAb levels in serum of aPAP (n = 110), sarcoidosis (n = 90), HP (n = 44), healthy controls (n = 31), sarcoidosis with complication of aPAP (n = 2) and HP with complication of aPAP (n = 1) patients. Horizontal dotted line shows the 95th percentile value for healthy controls (1.42 μg/mL) and horizontal solid line shows cut-off value (3.33 μg/mL) for discriminating aPAP from healthy controls. *GMAb* anti-granulocyte–macrophage colony-stimulating factor autoantibody, *aPAP* autoimmune pulmonary alveolar proteinosis, *HP* hypersensitivity pneumonitis

Serum GMAb levels in aPAP were significantly higher than those in healthy controls and sarcoidosis and HP patients (Fig. 2).

### Comparison of clinical features between patients with low-GMAb and elevated-GMAb sarcoidosis and HP

**Fig. 3:**
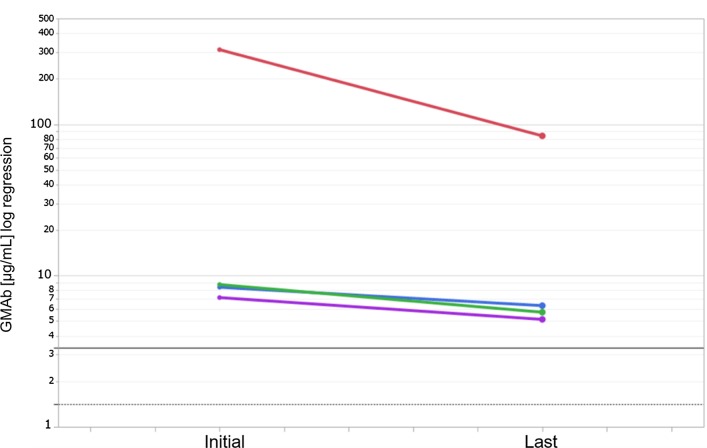
Changes in GMAb levels in four elevated-GMAb patients without the complication of aPAP. We measured serum GMAb at diagnosis and follow-up latest point in elevated-GMAb sarcoidosis (n = 2) and HP (n = 2) patients whose serum samples were obtained after diagnosis. Data at "initial" and "last" are the GMAb levels at the time of diagnosis and most recently after diagnosis, respectively. The median interval between measurements of GMAb levels was 4.4 years (IQR: 1.1, 7.8). Horizontal black dotted line shows the 95th percentile value for healthy controls (1.42 μg/mL) and horizontal black solid line shows cut-off value (3.33 μg/mL) for discriminating aPAP from healthy controls. *GMAb* anti-granulocyte–macrophage colony-stimulating factor autoantibody, *aPAP* autoimmune pulmonary alveolar proteinosis, *HP* hypersensitivity pneumonitis, *IQR* interquartile range

The number of elevated-GMAb sarcoidosis patients, including comorbid patients with aPAP (n = 2), was five (5.4%). The number of elevated-GMAb HP patients, including a comorbid patient with aPAP (n = 1), was five (11.1%). We also measured the serum GMAb levels of the most recent samples obtained after diagnosis from the four elevated-GMAb patients (two sarcoidosis and two HP patients) without complication of aPAP. The GMAb levels of all four patients remained higher than the cut-off values (Fig. 3). The median interval between measurements of GMAb levels was 4.4 years (IQR: 1.1, 7.8).

Clinical characteristics of 92 sarcoidosis patients, grouped according to elevated- or low-GMAb status, are shown in Table 2. Elevated-GMAb sarcoidosis patients presented with higher lactate dehydrogenase (LDH) (*p* = 0.035), KL-6 (*p* = 0.003), and SP-D (*p* = 0.022) levels than those of low-GMAb patients. More sarcoidosis patients with elevated-GMAb required systemic corticosteroid therapy after diagnosis than patients with low-GMAb (*p* = 0.002). Elevated-GMAb sarcoidosis patients without aPAP complication (n = 3) presented with higher KL-6 levels than those of low-GMAb patients (n = 87) (*p* = 0.049). Sarcoidosis patients with elevated-GMAb and without aPAP complication also required systemic corticosteroid therapy after diagnosis (n = 2, 66.7%) more frequently than low-GMAb patients (n = 10, 11.4%) (*p* = 0.048).

**Table 2:** Comparison of clinical features of low- and elevated-GMAb sarcoidosis patients

Characteristics	Low-GMAb (n = 87)	Elevated-GMAb (n = 5)	*p*-value
Age, years	58 [38, 70]	60 [46, 64]	0.849
Male, n	37 [42.5]	2 [40]	1.000
BAL Ly, %	27.6 [19, 40.8]	51.5 [33.6, 71.6]	0.073
BAL CD4/CD8	7.1 [0.82, 7.83]	7.1 [3.8, 8.2]	0.723
LDH, U/L	185 [159–205]	368 [321–414]	0.035
KL-6, U/mL	352 [242, 581]	2318 [871, 9608]	0.003
SP-D, ng/mL	64 [33, 115.7]	244 [80, 882]	0.022
SP-A, ng/mL	31.2 [24.6, 47.5]	47.2 [21.2, 135]	0.475
CEA, ng/mL	2.2 [1.4–3.1]	5.2 [1.9–8.5]	0.444
CYFRA, ng/mL	1.9 [1.2–2.8]	8.1 [5.4–10.7]	0.090
ACE, U/L	21.8 [16.8, 28.3]	31.3 [17.5, 40.8]	0.146
sIL-2R, U/mL	812 [522, 1380]	954 [608, 2011]	0.518
%FVC, %	101.3 [83.8, 114.4]	81.1 [76.2, 110]	0.406
%DLco, %	79.5 [63.8, 97.3]	53.5 [34.7, 103]	0.334
Extra pulmonary lesions, n	47 [54.0]	4 [80]	0.376
Eye, n	28 [32.2]	3 [60]	
Skin, n	6 [6.9]	1 [20]	
Heart, n	5 [5.7]	1 [33.3]	
Others, n	9 [10.3]	1 [33.3]	
Use of steroids, n	10 [11.5]	4 [80]	0.002

Data are expressed as the median [IQR] or number [%]

*GMAb* anti-granulocyte–macrophage colony-stimulating factor autoantibody, *BAL* bronchial alveolar lavage, *Ly* lymphocytes, *LDH* lactate dehydrogenase, *KL-6* Krebs von den Lungen-6, *SP-D* surfactant protein-D, *SP-A* surfactant protein-A, *CEA* carcinoembryonic antigen, *CYFRA* cytokeratin fragment 21-1, *ACE* angiotensin-converting enzyme, *sIL-2R* soluble interleukin-2 receptor, *%FVC* percent predicted forced vital capacity, *%DLco* percent predicted carbon monoxide diffusing capacity

Characteristics of 45 HP patients, grouped according to elevated- or low-GMAb status, are shown in Table 3. Patients with elevated-GMAb HP presented with older age (*p* = 0.039), higher lymphocyte counts in BAL (*p* = 0.019), and higher serum levels of KL-6 (*p* = 0.001), SP-D (*p* = 0.004), carcinoembryonic antigen (CEA) (*p* = 0.023), and cytokeratin fragment 21-1 (CYFRA) (*p* = 0.001) than did patients with low-GMAb HP. Whereas elevated-GMAb HP patients without aPAP complication (n = 4) presented with higher lymphocyte counts in BAL (*p* = 0.016), and higher serum levels of KL-6 (*p* = 0.003), and SP-D (*p* = 0.007) than those of low-GMAb subjects (n = 40).

**Table 3:** Comparison of clinical features of low- and elevated-GMAb HP patients

Characteristics	Low-GMAb (n = 40)	Elevated-GMAb (n = 5)	*p*-value
Age, years	59.5 [50.5–68.5]	71 [64.5–73.5]	0.039
Male, n	23 [57.5]	2 [40]	0.652
Chronic HP, n	35 [87.5]	5 [100]	1.000
*Trichosporon asahii*, n	5 [14.7]	1 [20]	1.000
BAL Ly, %	17.4 [8.1–79.9]	69.9 [47.8–81.1]	0.019
BAL CD4/CD8	1.7 [0.62–3.95]	2.1 [1.1–8.6]	0.437
LDH, U/L	208 [196–258]	236 [211–327]	0.213
KL-6, U/mL	1416 [797–2457]	4638 [3430–9674]	0.001
SP-D, ng/mL	180 [102–339]	491 [330–689]	0.004
SP-A, ng/mL	59.5 [40.1–96.3]	69.3 [51.0–150]	0.426
CEA, ng/mL	3.7 [2.4–6.2]	6.1 [5.6–16.1]	0.023
CYFRA, ng/mL	2.8 [2.3–4.4]	3.7 [2.9–5.3]	0.001
%FVC, %	83.1 [64.2–90.1]	95.5 [82.4–116.9]	0.108
%DLco, %	64.3 [55.1–79.1]	74.6 [56.6–79.5]	0.627
Use of steroids, n	15 [37.5]	4 [80]	0.146

Data are expressed as the median [IQR] or number [%]

*HP* hypersensitivity pneumonitis, *GMAb* anti-granulocyte–macrophage colony-stimulating factor autoantibody, *BAL* bronchial alveolar lavage, *Ly* lymphocytes, *LDH* lactate dehydrogenase, *KL-6* Krebs von den Lungen-6, *SP-D* surfactant protein-D, *SP-A* surfactant protein-A, *CEA* carcinoembryonic antigen, *CYFRA* cytokeratin fragment 21-1, *%FVC* percent predicted forced vital capacity, *%DLco* percent predicted carbon monoxide diffusing capacity

### Radiological and pathological re-evaluation in patients with elevated-GMAb sarcoidosis and HP patients

The radiological and pathological re-evaluation of findings related to PAP in elevated-GMAb sarcoidosis and HP patients without aPAP diagnosis is shown in Table 4. In elevated-GMAb sarcoidosis patients without aPAP, GGO (2 of 3 patients) and interlobular septal thickening (1 of 3 patients) were observed on CT scans. Two elevated-GMAb HP patients without aPAP presented with eosinophilic granular material, and two patients presented with foamy cells within lung alveoli.

**Table 4:** Radiological and pathological assessment in elevated-GMAb sarcoidosis and HP patients (GMAb ≧ 3.33 μg/mL) without aPAP complication

Disease	Sarcoidosis (n = 3)			HP (n = 4)			
GMAb (μg/mL)	4.43	6.33	311	4.34	7.16	8.72	8.72
Radiological findings							
GGO	+	+	−	+	+	+	+
Interlobular septal thickening	+	−	−	−	−	−	−
Crazing paving pattern	−	−	−	−	−	−	−
Consolidation	−	−	−	−	−	−	−
Geographic distribution	−	−	−	−	−	−	−
Subpleural sparing	−	−	−	−	−	−	−
Traction bronchiectasis	+	−	−	+	+	+	+
Cyst	−	+	−	+	+	+	−
Honeycombing	−	−	−	−	−	−	−
Pathological findings							
Eosinophilic, granular materials	−	−	−	−	−	+	+
PAS staining-positive materials	−	−	−	−	−	−	−
SP-A positive materials	−	−	−	−	−	−	−
Foamy macrophages	−	−	−	−	+	+	−
Hyaline globule/sclerotic central core	−	−	−	−	+	+	+
Lymphocytic infiltration	−	+	−	+	+	+	+
Interstitial fibrosis	+	+	+	+	+	+	+
Neoplastic lesion	−	−	−	−	−	−	−
Granulomatous lesion	+	+	−	+	+	+	+
Neutrophilic/eosinophilic infiltration	−	−	−	+	−	−	+
Necrosis	−	−	−	−	−	−	−

Serum GMAb levels were measured at sarcoidosis or HP diagnosis

*aPAP* autoimmune pulmonary alveolar proteinosis, *GMAb* anti-granulocyte–macrophage colony-stimulating factor autoantibody, *HP* hypersensitivity pneumonitis, *GGO* ground glass opacity, *PAS* periodic acid-Schiff, *SP-A* surfactant protein-A

## Discussion

To the best of our knowledge, this is the first study to clarify the incidence and clinical features of patients with elevated-GMAb sarcoidosis and HP. The percentages of elevated-GMAb sarcoidosis and HP patients were 5.4% and 11.1%, respectively. Of the five elevated-GMAb sarcoidosis patients, two were complicated with aPAP. Of the five elevated-GMAb HP patients, one was complicated with aPAP. Significantly higher serum levels of LDH, KL-6, and SP-D were noted in patients with elevated-GMAb sarcoidosis than in patients with low-GMAb sarcoidosis. Even in elevated-GMAb HP patients, significantly higher serum levels of KL-6, SP-D, CEA, CYFRA, and higher lymphocyte count in BAL were observed than in low-GMAb patients. These clinical features observed in elevated-GMAb sarcoidosis and HP patients are considered characteristic of patients with aPAP. Further, elevated-GMAb sarcoidosis and HP patients without aPAP complication presented with some radiological and pathological features characteristic of aPAP. We diagnosed patients with aPAP if both pathological and radiological findings were consistent with the features of PAP, in addition to having GMAb levels higher than 95th percentile of healthy controls. Elevated-GMAb sarcoidosis and HP patients without aPAP complication did not meet all these criteria and were not diagnosed with aPAP before the present study; however, subclinical aPAP or early-stage aPAP was suspected.

There are several reports on GMAb cut-off values for aPAP diagnosis. Using polyclonal GMAb as the standard, Nishimura et al. [20] and Nakata et al. [29] reported cut-off values of 2.8 µg/mL and 1.0 µg/mL for GMAb, respectively. By comparison, using monoclonal GMAb as the standard, we reported a cut-off value of 0.5 µg/mL [2], whereas Uchida et al. considered 5.0 µg/mL as the cut-off value [27]. The cut-off value (3.33 µg/mL) that we reassessed in this cohort study using monoclonal GMAb was appropriate compared to previous GMAb reports.

Most of the previous studies have reported GMAb cut-off values for both aPAP and healthy controls [6, 27]. Recently, Nakata et al. validated a new serum GMAb testing kit and provided an optimal cut-off value for these individuals [29]. Several studies have evaluated GMAb levels in other lung diseases [5, 8, 20]. Nishimura et al. demonstrated the cut-off serum GMAb values measured by ELISA to discriminate aPAP from healthy controls or other lung diseases as 2.0 μg/mL (sensitivity 100%, specificity 100%) or 2.8 μg/mL (sensitivity 100%, specificity 98%) [20]. They reported that GMAb levels in sarcoidosis and pneumoconiosis patients were slightly higher than those in healthy controls [20]. In our study, some sarcoidosis and HP patients showed higher GMAb levels than healthy controls. We conducted ROC curve analysis for differentiating aPAP from the following combinations: (1) healthy controls, (2) sarcoidosis and HP patients, and (3) healthy controls, sarcoidosis, and HP patients. The GMAb cut-off value was 3.33 µg/mL for discrimination by aPAP for all the three aforementioned combinations.

Several aPAP cases complicated with sarcoidosis have been reported [14–17]. Environmental exposure to microbial agents, including *Mycobacterium* and *Propionibacterium acnes,* may be the causative factors of sarcoidosis [30, 31]. Infiltration of macrophages, T-cells, and cytokines such as GM-CSF results in granuloma formation [30, 31]. Patients with aPAP are immunocompromised, as macrophages and neutrophils become dysfunctional due to GMAb [32, 33]. They are susceptible to infection and could present with sarcoidosis as a result of exposure to microbial agents. However, sarcoidosis preceding aPAP has also been reported [17, 18]. Akasaka et al. reported that corticosteroid therapy may worsen the disease severity in aPAP patients because corticosteroids suppress the function of macrophages [34]. In patients with sarcoidosis preceding aPAP, corticosteroid therapy for sarcoidosis could worsen early-stage or subclinical aPAP and result in aPAP complications. Verma et al. reported five patients with histopathological features of PAP and HP on surgical lung biopsy, although they did not measure GMAb [19]. Environmental factors may trigger aPAP as well as HP [13]. Infection in aPAP patients could be related to HP pathogenesis. On the contrary, in HP, lipoproteinaceous materials could accumulate after increased leakage to the alveolar spaces. Further studies are required to clarify these possibilities and whether sarcoidosis/HP or aPAP preceded the other, or if they occurred simultaneously.

Measuring GMAb values at the diagnosis of sarcoidosis or HP is desirable to enable detection of coexisting subclinical or early-stage aPAP. In sarcoidosis, more patients with elevated-GM-Ab required systemic corticosteroid therapy than patients with low-GMAb. Two cases of aPAP complicated with sarcoidosis in this study were previously reported and also required systemic corticosteroid therapy for sarcoidosis [18]. We reduced the corticosteroid dose in two patients to prevent aPAP aggravation.

On CT, findings of HP and aPAP are often difficult to distinguish. However, aPAP complication should be considered if sarcoidosis patients have GGO or interlobular septal thickening on CT scans. Pathological re-evaluation revealed features suggestive of aPAP in HP patients. It is important to evaluate the aPAP pathology in these patients alongside GMAb measurements.

The major limitations of the current study are the small sample size and retrospective design because of the rarity of the disease and the associated comorbidity. Studies with a larger sample of patients with varying diseases and disorders are required to determine the optimal cut-off value of GMAb and the incidence of increased level of GMAb in sarcoidosis and HP.

## Conclusions

We demonstrated the percentage of elevated-GMAb sarcoidosis and HP patients who presented with several features suggestive of aPAP. In elevated-GMAb sarcoidosis and HP patients without aPAP diagnosis, subclinical or early-stage aPAP might be present and may not necessarily indicate false positives. Upon diagnosis of sarcoidosis or HP, GMAb should be measured to detect the possible comorbidity of subclinical or early-onset aPAP. Further longitudinal studies are required to determine the potential prognostic role of GMAb measurement in sarcoidosis and HP patients.

## Data Availability

The datasets used and/or analyzed during the current study are available from the corresponding author on reasonable request.

## Abbreviations

aPAP: Autoimmune pulmonary alveolar proteinosis
BAL: Bronchoalveolar lavage
CT: Computerized tomography
ELISA: Enzyme-linked immunosorbent assay
GGO: Ground glass opacity
GMAb: Anti-granulocyte–macrophage colony-stimulating factor autoantibody
GM-CSF: Granulocyte–macrophage colony-stimulating factor
HP: Hypersensitivity pneumonitis
HRCT: High-resolution computed tomography
PAS: Periodic acid-Schiff
ROC: Receiver operating curve
SP: Surfactant protein
